# Just add water: Rainfall‐induced anther closure and color change in *Ripariosida hermaphrodita* (Malvaceae)

**DOI:** 10.1002/ece3.10219

**Published:** 2023-07-03

**Authors:** Emily A. Humphreys, Cynthia Skema

**Affiliations:** ^1^ Morris Arboretum & Gardens of the University of Pennsylvania Philadelphia Pennsylvania USA

**Keywords:** anther closure, floral color change, Malvaceae, pollination, rainfall

## Abstract

Anther opening has commonly been thought of as unidirectional, but reports of anthers closing in response to rainfall show this is not the case. In some species, anther closure can protect pollen from degrading or washing away, thus possibly enhancing male fitness. Similarly, although floral color is often presumed to be static, numerous floral parts may change color during blooming. These color changes primarily occur in response to pollination or aging, thus potentially increasing pollination efficiency by directing floral visitors to recently opened, unpollinated flowers. Daily observations of 364 *Ripariosida hermaphrodita* flowers from seven individuals showed that anthers that were purple, open, and shedding pollen became beige colored and tightly closed after rainfall. These findings were further supported by observations of plants exposed to simulated rainfall in a greenhouse and time‐lapse photography of flowers misted with water. To our knowledge, our work represents the first report of anther closure in response to rain in Malvaceae and the first report of floral color change induced by rainfall.

## Graphical abstract

Daily observations, an experimental greenhouse trial, and time‐lapse photography of *Ripariosida hermaphrodita* flowers showed that anthers that were purple, open, and shedding pollen became beige colored and tightly closed after rainfall. To our knowledge, our work represents the first report of anther closure in response to rain in the Cotton Family (Malvaceae) and the first report of floral color change induced by rainfall.

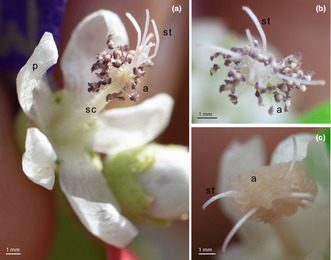

## 1 INTRODUCTION

Abiotic factors, such as temperature, wind, and rain, have the potential to impact pollination success. In animal pollinated flowers, they do this both by influencing pollinator behavior and by impacting floral structures directly (Goldblatt & Manning, 2006; Hennessy et al., 2021; Shrestha et al., 2018). As such, dynamic responses to environmental cues may increase a plant's reproductive fitness (Goldblatt & Manning, 2006; Shrestha et al., 2018). For example, lotus (*Nelumbo nucifera*) flowers maintain a steady range of floral receptacle temperatures, as a reward for pollinators, by increasing heat production when experiencing low ambient temperatures (Seymour et al., 1998). In response to the potential of high‐winds to damage structures, daffodils (*Narcissus*) have been shown to reorient flowers (Gardiner et al., 2016). Similarly, to reduce the potential of rain to dilute nectar, wash away pollen, and decrease pollen viability, numerous floral adaptations have evolved, such as pendulous flowers, petals fused into a tubular shape, protective hairs, and dynamic floral closure (Aizen, 2003; Bynum & Smith, 2001).

### 1.1 Anther closure

Anther closure after primary anther dehiscence, a phenomenon which has also been called reversible anther opening (Edwards & Jordan, 1992), is the floral closure response to rainfall that is of interest to our study. Though anther opening is commonly assumed to be a unidirectional process (Li et al., 2012), there are at least 17 families with species that close their anthers in response to rain, across gymnosperms, magnoliids, monocots, and eudicots (Cruden, 1973; Edwards & Jordan, 1992; Kerner von Marilaun, 1895; Li et al., 2012; Pacini et al., 2014; Wang et al., 2009). Studies have shown that anther closure after rain in some species has the potential to prevent pollen from washing away and enhance pollen longevity, and when accompanied by subsequent anther reopening is thought to enhance male fitness (Edwards & Jordan, 1992; Li et al., 2012). Despite its presence and potential fitness implications, this phenomenon has been largely forgotten; the majority of reports of anther closure after rain come from Kerner von Marilaun's narrative compilation from over a century ago (1895).

### 1.2 Anther color change

Color change in flowers in prime bloom is a relatively widespread phenomenon seen in more than 20% of families and at least 456 species (Weiss & Lamont, 1997). Most flowers change color as they age or upon pollination (Ruxton & Schaefer, 2016; Weiss & Lamont, 1997). Abiotic factors including light (Farzad et al., 2002; Jabbari et al., 2013; Yan et al., 2017) and temperature (Larson & Barrett, 1999) have also been found to modulate floral color change. Studies have shown that many pollinators preferentially visit pre‐change flowers, which may increase pollination efficiency and rates of outcrossing (Ruxton & Schaefer, 2016; Weiss & Lamont, 1997). At the same time, producing and altering pigments can be energetically costly and the benefits of floral color change may be dependent on variable spatio‐temporal conditions (Ruxton & Schaefer, 2016).

Color change in anthers has been less commonly reported than color change in other floral parts (Weiss, 1995; Weiss & Lamont, 1997). In reporting on 393 species with floral color change, Weiss (1995) outlined 10 floral parts which change color; anthers were not among these parts (though filaments were considered). In their later review paper, Weiss and Lamont (1997) identified 456 species with floral color change of which only one, a member of *Calectasia*, displayed anther color change. Our literature review found some degree of anther color change has been observed in the 15 species comprising the genus *Calectasia* (Dasypogonaceae; Barrett & Barrett, 2015; Barrett & Dixon, 2001), as well as eight species of *Salix* (Salicaceae; Smith, 1945), *Crinum joesmithii* (Amaryllidaceae; Barrett & Barrett, 2015), *Paepalanthus bifidus* (Eriocaulaceae; Martins Junior et al., 2022), *Rhexia virginica* (Melastomataceae; Larson & Barrett, 1999), and *Rosa rugosa* (Rosaceae; Dobson et al., 1999). None of these instances of anther color change were in response to rainfall.

### 1.3 Study species

**FIGURE 1:**
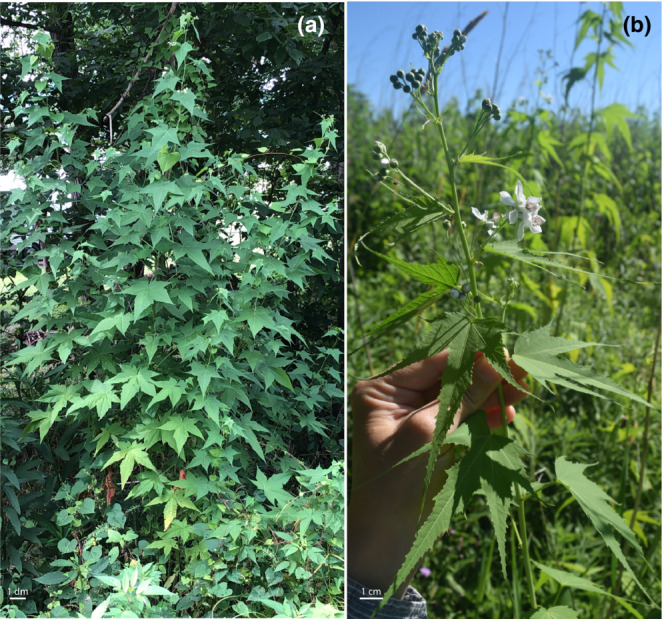
(a) Whole plant image of *Ripariosida hermaphrodita* in the wild. (b) Inflorescence of *R. hermaphrodita*. Images by C. Skema.

*Ripariosida hermaphrodita* is an herbaceous perennial, native to eastern North America (Spooner et al., 1985; Thomas, 1979). Its small, white flowers appear to be pollinated by generalists; we have observed Hymenoptera, Diptera, and Lepidoptera visiting the flowers (E. A. Humphreys and C. Skema, personal observations). Belonging to the monospecific genus *Ripariosida*, *R. hermaphrodita* is phylogenetically, morphologically, and geographically distinct from its closest relatives (Weakley et al., 2017). *R. hermaphrodita* has been ranked as globally vulnerable and is imperiled or critically imperiled in Indiana, Kentucky, Maryland, Ontario, Pennsylvania, and Virginia (NatureServe, 2022). Growing as tall as four meters in a single season (Figure 1; Weakley et al., 2017; C. Skema, personal observations), there is European interest in *R. hermaphrodita's* use as a bio‐fuel crop (as reviewed in Nahm & Morhart, 2018). Despite *R. hermaphrodita's* rarity and distinctive characteristics, relatively little is known about its fundamental biology and reproduction.

Here, we report novel observations of rainfall‐induced anther closure and color change in *R. hermaphrodita*. Anther closure in response to rain has not previously been reported in Malvaceae. To our knowledge, rainfall‐induced floral color change has never before been observed. Together, these findings begin to characterize this rare plant's reproductive biology and provide new insight into how dynamic abiotic factors may affect floral evolution.

## 2 METHODS

### 2.1 Floral observations

We present data from seven *R. hermaphrodita* plants that were observed from July 11 to September 8, 2022. The plants were grown from seed, potted up, and maintained outdoors, with regular watering. The seed originated from three wild‐collected populations and one cultivated source of unknown provenance. Flowers were observed once daily between 5:45 a.m. and 1:45 p.m. (EDT), with the exception of July 24 and 25, on which days no observations were made. Each day, the color of the anthers was recorded as well as the position of the anthers (open or closed). Beginning August 11, we also recorded whether the anthers were visibly covered with a film of rainwater. To aid in observation, 10× and 20× hand lenses were used.

To be included in our analyses, floral data had to be “complete,” meaning they met the following criteria: at least one observation was made of the flower before the petals had expanded enough to reveal the androecium, daily observations were recorded while the flower was in bloom, and a final observation indicated the flower had senesced, here defined as the abscission of the basally fused unit of the corolla + androecium. Data were rendered incomplete for many reasons, including the loss of a label, lateral shoot damage, floral herbivory, and missed days of observations.

### 2.2 Natural rainfall data

Rainfall records were obtained through National Weather Service's NOWData (National Weather Service, 2022). Data for “Philadelphia Area, PA” were downloaded on October 12, 2022. These data were recorded at Philadelphia International Airport which is roughly 16 km from the study site. For days where precipitation was present but quantities were too small to measure, these records reported “Trace” rainfall. We treated “Trace” rainfall as 0 cm. We define a “rain event” as a date or continuous string of dates with greater than “Trace” rainfall. Initially, direct observations of rain events at the study site were not recorded because we were not anticipating *R. hermaphrodita* would display rainfall responses when the observation protocol was designed.

### 2.3 Simulated rainfall experiment

We conducted a simulated rainfall experiment on September 13, 2022. Six plants of *R. hermaphrodita* were housed in a greenhouse and watered only at the soil level to ensure the flowers in anthesis had not been exposed to water. On each plant, the lowest six flowers that met experimental criteria were observed. These criteria required flowers to be in prime bloom, have pollen on their anthers, be free from damage, and be low enough on the plant that water falling from our overhead watering system would reach them. One plant had only three flowers that met these criteria, so only these three were included in experimentation. The six plants were made up of three pairs, with each pair representing plants grown from seed from the same population. One plant from each pair was chosen at random to be in the experimental group with the other being in the control group. For each flower, anther color and position were recorded. Then the three plants selected for experimental treatment were watered for 30 min with an overhead watering system. Immediately after watering, observations were repeated. One flower in the experimental group was lost over the course of experimentation and observation, bringing the control group to 18 flowers and the experimental group to 14 flowers. Observations were made over the course of 2 h. We alternated between observing experimental and control flowers, so that neither group was, on aggregate, observed first. The four additional control flowers were observed at the end of the observation period.

### 2.4 Data analysis

All observations of anther position in *R. hermaphrodita* flowers were assigned to three categories: “closed” if most anthers were closed; “open” if most anthers were open; or “mixed” if there was a nearly even mix of open and closed anthers or anther position could not be determined. Similarly, observations of anther color were assigned to three categories: “purple” if most anthers were purple; “beige” if most anthers were translucent, white, beige, yellow, or brown; or “mixed” if there was a nearly even mix of purple and beige anthers, the anthers were an intermediate purple‐beige, or anther color could not be determined.

R Studio (v. 4.2.0) was used to analyze and visualize all data (RStudio Team, 2022) with the exception of finishing details (arrows, etc.) which were added to graphs in Adobe Illustrator (v. 27.2) and Adobe Photoshop (v. 24.2.0). To better understand the results of the simulated rainfall experiment, R Studio's “chisq.test” function was used to perform Pearson's Chi‐squared test (RStudio Team, 2022). This Chi‐squared test with Yate's continuity correction was used to test for statistically‐significant differences in anther color and position change in the flowers of the experimental group compared to those of the control group. So few anthers showed greater pigmentation or a more‐closed anther position that these data would have rendered these Chi‐squared tests unreliable; therefore, they were omitted in both the experimental and control group. A second Chi‐squared test was used to compare anthers before and after treatments for the simulated rainfall data. For the natural rainfall data, standard error was calculated for average cumulative rainfall totals and used to compare rain events associated and not associated with transitions in anther color and position across >88% of flowers.

### 2.5 Time‐lapse photography

Multiple series of photographs were taken of the anthers of *R. hermaphrodita* flowers and combined into time‐lapse videos. These flowers were sprayed with water to simulate a rain event. Photographs were taken with a Canon EOS 5DS camera connected to a Zeiss Stemi 2000‐C microscope at a magnification that allowed anthers to fill, but not exceed, the frame (approximately 25× magnification). Flowers were clipped from potted plants and brought to the microscope area. To improve visibility, sufficient anthers were trimmed from flowers to leave a (roughly) single horizontal plane of anthers. If an abundance of pollen obscured the view of the remaining anthers, some pollen was removed to reveal more of the anther walls.

Variables such as time‐lapse length, number of photos, magnification, video angle, and frequency of water application were manipulated to determine what combination best showed the phenomenon of interest. Ultimately, two time‐lapse series capturing anther closure and color change were created. Video S1 was made up of 450 photos, with the camera shooting a photo every 3 s, for a total time frame of 22:30 min. For the first 11 min of imaging, the anthers were sprayed with tap water every 5 s using a small spray bottle. Video S2 was made up of 450 photos, with the camera shooting a photo every 9 s, for a total time frame of 67:30 min. For the first 10 min, the anthers were sprayed with tap water every 5 s. A time counter was added to each time‐lapse video in iMovie (v.10.3.5).

## 3 RESULTS

### 3.1 Floral observations and natural rainfall

**FIGURE 2:**
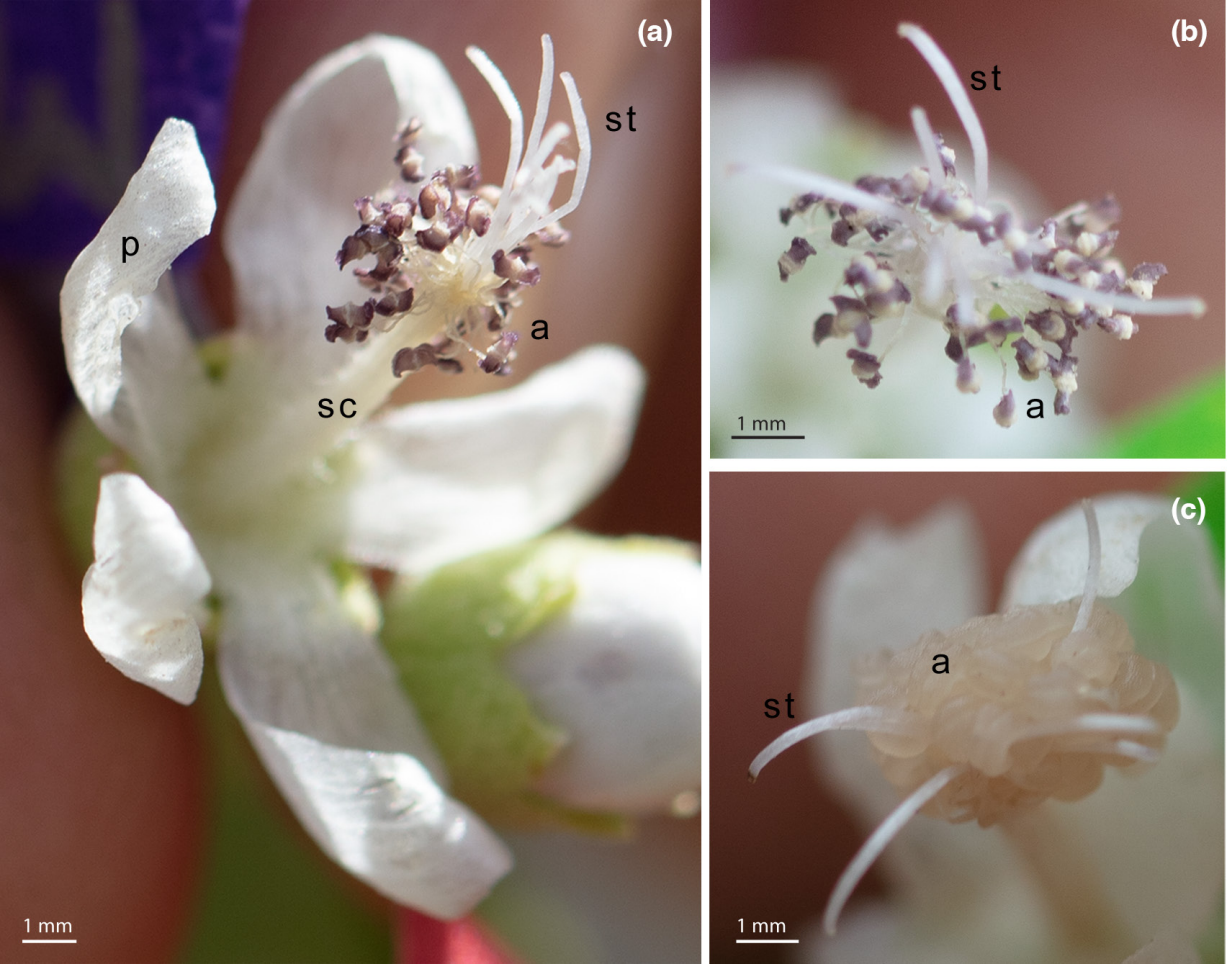
(a) Floral morphology of *Ripariosida hermaphrodita*. (b) The open, purple anthers of flower #360 on August 21, 2022. There was no rain during the week before this date. (c) The closed, beige anthers of flower #360 on August 22, 2022, after a rain event. a, anthers; p, petals; sc, staminal column; st, style lobes. Images by Emily A. Humphreys.

Observations of anthesis—focused on androecial maturation, anther dehiscence, and pollen shedding—were recorded for 542 flowers total over the course of our 60‐day study. Of these, records for 364 flowers (67%) were “complete” (as defined in Methods) and, thus, included in our analyses. The general progression for androecial development in flowering *R. hermaphrodita* was as follows: petals expand enough to reveal the androecium, then within minutes the petals unfurl and most to all of the anthers open and begin shedding pollen (Figure 2a). One day later, most pollen has been shed. On average, the flowers senesce 3 days after the androecium is revealed. Throughout the flowering period, anthers are typically open and purple (Figure 2b). Because of the short span of time between the androecium being revealed and the anthers opening, very few flowers were observed during this period.

**FIGURE 3:**
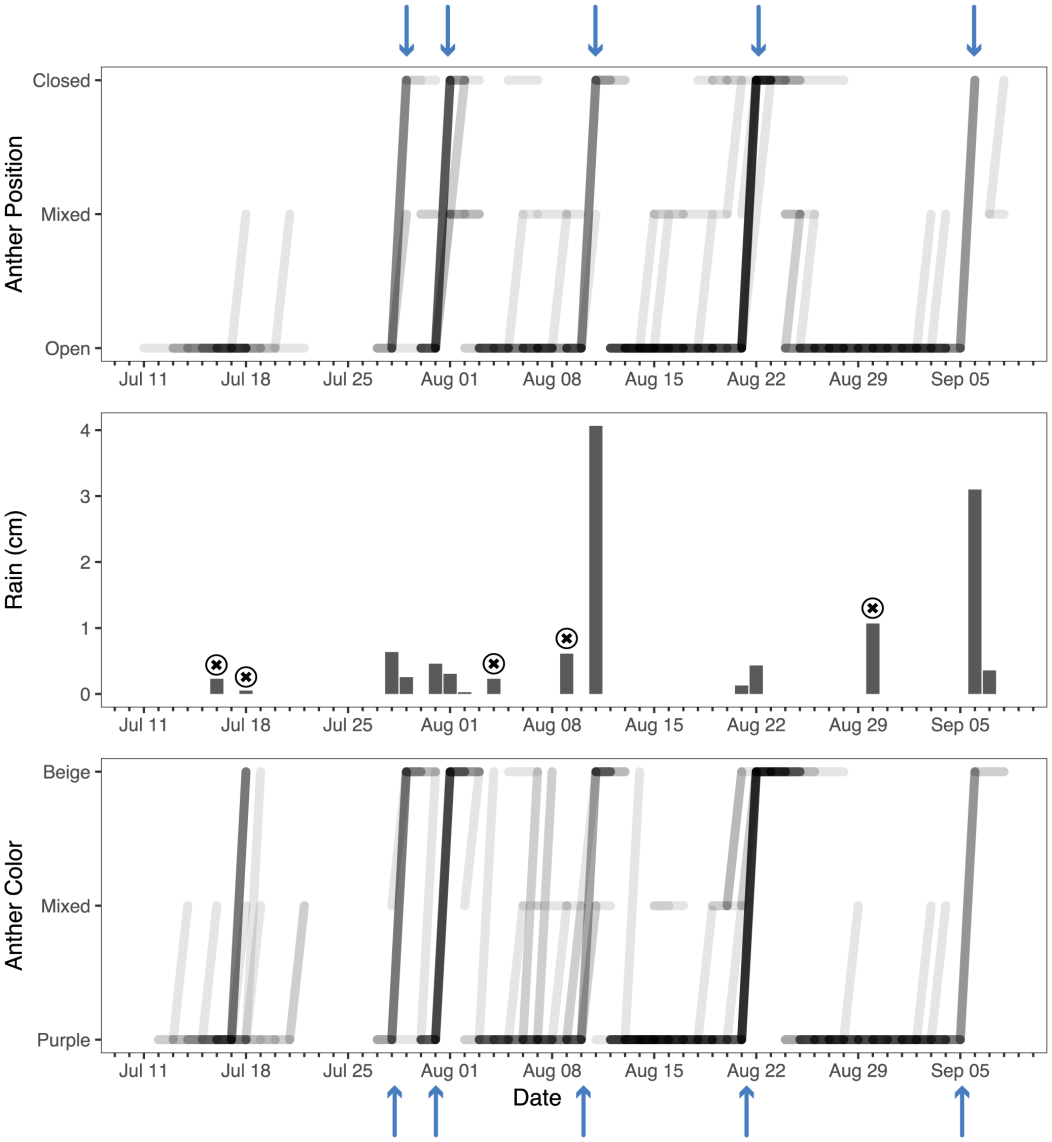
Changes in anther position (top row) and color (bottom row) away from open/purple and towards closed/beige in *Ripariosida hermaphrodita* corresponded to rainfall events (middle row). Each line connects points from an individual flower and is 90% transparent. As such, more opaque lines indicate a greater number of flowers on the same trajectory. Circled Xs indicate rain events with no corresponding response in >50% of flowers. Blue arrows indicate transitions in anthers in >88% of flowers.

Field observations highlighted a remarkable change in *R. hermaphrodita* anthers following rain events. After rainfall, anthers appeared tightly closed, swollen, and beige, white, or translucent in color (Figure 2c). There were five dates when anther position transitioned to a more‐closed state in more than 88% of flowers (Figure 3, top row, blue arrows). Each of these transitions occurred the same day as a rainfall event or the day after. Five rain events did not correspond to a transition in anther position from open to closed (circled Xs).

Similarly, anther color transitioned to less pigmented (more beige) in more than 88% of flowers on the same five dates (Figure 3, bottom row, blue arrows). All of these transitions occurred the same day as a rainfall event or the day after. Five rain events did not correspond to a broad transition in anther color across >50% of flowers (Figure 3, circled Xs). The rain event on July 18 had the smallest rainfall total of any event observed at 0.05 cm; it corresponded to less pigmented (more beige) anthers across 50% of flowers.

Our data show only one flower demonstrated anther reopening directly (from closed to open, without mixed intervening) and none regained anther color directly (beige to purple). With such small numbers, these results are not informative and we conclude that anther closure and loss of pigmentation are likely not reversable. Rain events that corresponded to transitions in anther position and color had an average cumulative precipitation of 1.95 ± 0.75 cm. Those that did not (Figure 3, circled Xs) had an average cumulative precipitation of 0.44 ± 0.18 cm. After we began recording if anthers were covered with a film of water on August 11, we observed most flowers to have covered anthers on four dates: August 11, August 22, August 23, and September 6. Each of these dates corresponded to a transition in anther color and position.

### 3.2 Simulated rainfall experiment

Over the 30 min of simulated rainfall, a rain gauge in the watering area collected 0.9 cm of water (±0.15 cm estimated measuring error). After exposure to the simulated rainfall treatment (experimental group), 93% of flowers had more‐closed anthers and 7% of flowers had anthers with no change in position. With no simulated rainfall exposure (control group), 11% of flowers had more‐closed anthers and 89% of flowers had anthers with no change in position. The difference between the experimental and control groups was significant (X^2^ = 17.98, df = 1, *p* = 2.2 × 10^−5^; data not shown).

Similarly, after exposure to the simulated rainfall treatment, 57% of flowers had lighter colored anthers and 43% of flowers had anthers with no change in pigmentation (including some that were beige before the simulated rainfall). In the control group, 11% of flowers had lighter colored anthers, 6% of flowers had more pigmented anthers, and 83% of flowers had anthers with no change in pigmentation. The difference between the experimental and control groups was significant (X^2^ = 5.31, df = 1, *p* = .021; data not shown).

**FIGURE 4:**
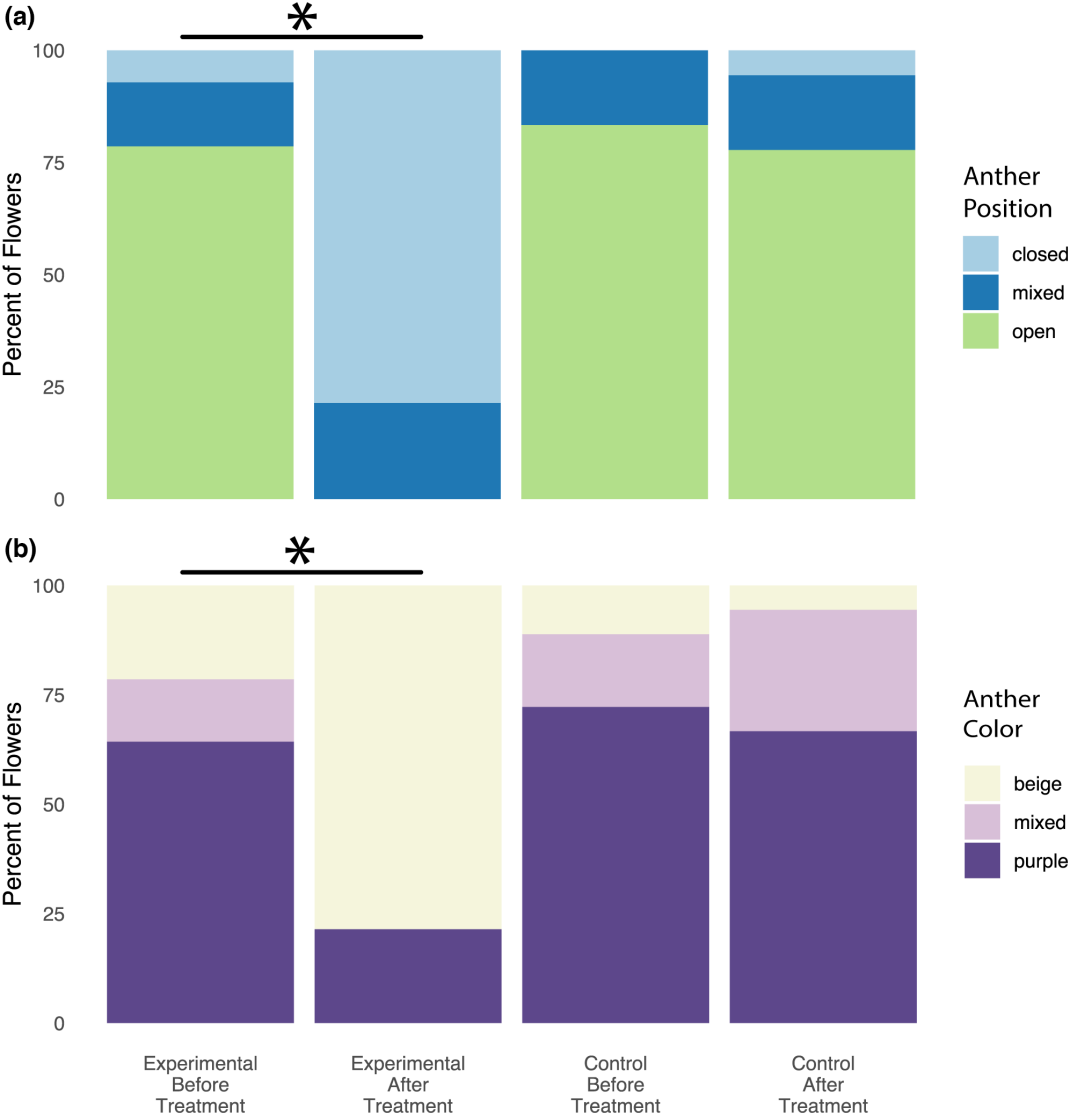
Simulated rainfall induces anther closure and color change in *Ripariosida hermaphrodita*. (a) Anther position and (b) color before and after simulated rainfall treatment. The differences between the experimental (14 flowers) and control (18 flowers) groups for both anther position and anther color were shown to be statistically significant. Asterisks indicate a *p*‐value of <.01.

Changes in the experimental and control groups, as a whole, were also compared (Figure 4). After treatment, the anther position and color totals changed significantly in the experimental group, with anthers closing and losing pigmentation (Position, X^2^ = 19.53, df = 2, *p* = 5.7 × 10^−5^; Color, X^2^ = 9.57, df = 2, *p* = 8.3 × 10^−3^), while those in the control group did not (Position, X^2^ = 1.04, df = 2, *p* = .60; Color X^2^ = 0.87, df = 2, *p* = .65).

### 3.3 Time‐lapse photography

We took time‐lapse series to document the changes in *R. hermaphrodita* anthers following water exposure. In our videos, water droplets caused anther walls to swell rapidly and approximately double in size as they bent inwards towards their line of dehiscence, enveloped pollen, and finally closed completely as they reached terminal size (Videos S1 and S2). This occurred after 5–10 min of intermittent water exposure. Anther depigmentation occurred more slowly and—in contrast to anther position—continued after anthers reached their terminal size. At the end of Video S1, which was shot over 22:30 min, anthers were lighter in color, but still tinted purple. At the end of Video S2, which was shot over 67:30 min, anthers were fully translucent.

## 4 DISCUSSION

### 4.1 Floral observations and natural rainfall

For half of the rain events (5 of 10), we observed associated transitions in anther color and position across >88% of flowers (Figure 3, blue arrows). Average rainfall was more than four times higher for rain events with associated anther change than for rain events without associated changes. Given this, it seems likely that larger amounts of rain produce a stronger, more consistent response in anthers. With the weather station roughly 16 km away from our study site, it is possible that a precipitation event, especially a small one, could pass over one location and not the other or that rainfall intensity at the two locations could vary. We hypothesize this is why we did not see floral change associated with every rain event recorded at the weather station. While this is a limitation for our natural rainfall data, the prevailing trends in these data—that *R. hermaphrodita* anthers close and lose color after rain—are further substantiated by the results of our simulated rainfall experiment and time‐lapse photography.

From August 11 onward, we noted whether the anthers we observed were covered with water, giving us a directly observed indicator for rainfall at the study site. There were three events where most of the anthers were enveloped in a film of water. These events correspond exactly with the three broad transitions in anther color and position observed during that period. This close association lends further support to the idea that differences in weather at the study site versus the weather station explain the five events without correlated anther closure and color change.

### 4.2 Anther closure

When anthers open, the final outward bending of the anther walls is often facilitated by dehydration (Keijzer, 1987; Keijzer et al., 1996; Wilson et al., 2011). This dehydration can be the result of evaporation, reabsorption, or both (Timerman & Barrett, 2021), and, in some cases, may be related to the sudden drop in relative humidity that anthers experience upon floral anthesis (Keijzer et al., 1996). This may explain why we observed *R. hermaphrodita* anthers opening immediately after buds opened and petals unfurled. In *Laurus nobilis*, *Lilium philadelphicum*, and *Butomus umbellatus*, rainfall has been suggested to lead to the rehydration of various anther tissues that then cause anthers to reclose (Edwards & Jordan, 1992; Li et al., 2012; Pacini et al., 2014). These tissues include valve cells (Pacini et al., 2014), the epidermis (Edwards & Jordan, 1992; Li et al., 2012), and the endothecium (Edwards & Jordan, 1992). In at least one case, this process occurs passively (Pacini et al., 2014). It is likely a similar mechanism that underlies anther closure in *R. hermaphrodita*.

Some plants have been known to reopen closed anthers, including *Mirabilis nyctaginea* (Cruden, 1973), *Butomus umbellatus* (Li et al., 2012), and *Paris polyphylla* var. *yunnanensis*, the last of which, in addition to closing anthers in response to rain, closes its anthers every night and reopens them in the morning (Wang et al., 2009). In *Lilium philadelphicum*, which has anthers that close during rainfall, it is thought that pollen viability extends through floral senescence (Edwards & Jordan, 1992) and for *Butomus umbellatus*, it has been shown that anther closure during rain extends pollen viability (Li et al., 2012). In contrast, *R. hermaphrodita* anthers do not reopen after rainfall and thus their closure can provide little male fitness advantage to the species. This is further supported by the relatively short flowering period of any single flower (an average of 3 days), the dispersal of most pollen in the day after initial anther opening, the large number of flowers per individual (Figure 1b), and the plant's long flowering season (Humphreys, personal observations). Given these reasons, it is likely that anther closure in response to rain may be a mere mechanical consequence of how anthers dehisce in *R. hermaphrodita* as opposed to a trait for which there is active selection. Evidence for this in other species may lie in displays of incomplete anther closure after rainfall (*Hemerocallis fulva*, *Clintonia borealis*; Edwards & Jordan, 1992) as they can likewise be assumed to have limited fitness benefit.

Though anther closure may not always be adaptive, this does not mean it is evolutionarily insignificant; traits with limited fitness benefit may eventually serve as pre‐adaptations for transitions in niche or life history strategy, rendering what were once physiological byproducts as valuable contributions to reproductive success (Walck & Hidayati, 2004). Future research could provide fascinating insight into whether rainfall‐induced anther closure predates shifts towards traits that would render pollen protection particularly valuable, such as fewer flowers, longer flowering time, or rainier niches.

In the past century, anther closure after rainfall has been little studied. To our knowledge, since Kerner von Marilaun's compilation (1895), only four additional species have been noted as displaying rainfall‐induced anther closure (Cruden, 1973; Edwards & Jordan, 1992; Li et al., 2012; Wang et al., 2009). In this report, we add a fifth. By and large, modern methods have not been used to compare anther closure across species or investigate the extent to which evolutionary pressures are behind this phenomenon, leaving a significant gap in the literature.

### 4.3 Anther color change

Rain events minimize the visual contrast of *R. hermaphrodita* flowers, which may impact insect foraging. With the exception of the anthers and occasionally the stigmas, all visible parts of *R. hermaphrodita* flowers above the calyx are white (Figure 2a). As such, the purple anthers create a visual contrast between the reproductive structures and the corolla. Floral color contrasts can allow pollinators to better identify desirable target flowers (Dafni et al., 1997; Van der Kooi et al., 2019). After a rain event, anthers in *R. hermaphrodita* change from purple to beige, reducing this visual contrast, and the flowers largely maintain this lack of contrast through floral senescence (Figure 3). Though our observations are limited to human perceptions of color, which are in some ways different from insect perceptions (Weiss & Lamont, 1997), this loss of contrast may preferentially direct pollinators to *R. hermaphrodita* flowers that have not undergone a rain event. This is notable as rainfall can degrade pollen and dilute nectar, leaving post‐rainfall flowers with less to offer pollinators and less potential to bring plants reproductive success (Lawson & Rands, 2019). As such, rainfall may be an advantageous facultative trigger for floral color change from the perspective of both plant and pollinator—providing an easy visual cue for decreases in the “usefulness” of a flower, at least in terms of pollen availability, and allowing for potential optimization of pollinator foraging behavior. Now that it has been established that floral color change after rainfall is possible, we encourage researchers to look for further examples of this phenomenon.

Still, the exact consequences of this anther color change on the pollination and reproductive fitness of *R. hermaphrodita* remains to be studied. For instance, we do not know how visible the purple anther walls are to pollinators, given heavy pollen loads potentially obscuring them from view, as well as the natural perceptions of colors/contrasts by these insects. Furthermore, if *R. hermaphrodita* is indeed pollinated by generalists, as we have observed, there is the potential that the effects of anther color change could be complicated by multiple pollinators responding to different floral cues, for example, the attraction of flies to beige/white floral parts versus bees to purple/blue floral parts (Richards, 1997). Therefore, pollinator preferences could enact different, and perhaps contradictory, selective forces on the flowers in this species. Furthermore, for a full picture of the potential push and pull of reproductive evolutionary pressures on *R. hermaphrodita*, other factors must also be considered, such as the timing and volume of nectar production (i.e., another pollinator reward besides pollen), as well as the timing and duration of stigma receptivity (i.e., female fitness), which appears to occur towards the end of pollen release (Humphreys, personal observations).

There are three main classes of pigments associated with floral color change: betalains, carotenoids, and anthocyanins (Weiss & Lamont, 1997). In plants, betalains are exclusively found in the order Caryophyllales (Grotewold, 2006), so this class is not relevant for *R. hermaphrodita*. While carotenoids create yellow and orange floral hues, anthocyanins create orange, red, purple, and blue colors (Grotewold, 2006). Anthocyanins are water soluble and changes in pH affect anthocyanin color and create not only shifts between red and blue, but also colorless compounds (Enaru et al., 2021; Grotewold, 2006; Weiss & Lamont, 1997). Given all these factors, we hypothesize that the transition from purple to beige observed in *R. hermaphrodita* anthers is most likely the result of a rainfall‐induced pH change or a loss of anthocyanins. Further investigation is needed to determine the exact mechanism of this observed color change.

## 5 CONCLUSION AND FUTURE WORK

We found that two unusual responses to rainfall co‐occur in *Ripariosida hermaphrodita*: anther closure and anther color change. Based on our literature review, this represents the first report of rainfall‐induced anther closure in Malvaceae and the first ever documentation of rainfall‐induced floral color change. Together, these findings shed light on complex floral processes with the potential to impact plant‐pollinator interactions. The evolutionary history underlying the development of these traits, the mechanisms of these responses, and their potential impacts on fitness are yet to be explored in *R. hermaphrodita*. More broadly, we hope our findings will spur renewed interest in anther closure and pave the way for investigation into rainfall‐induced floral color change.

## AUTHOR CONTRIBUTIONS

**Emily A. Humphreys:** Conceptualization (equal); formal analysis (lead); investigation (lead); methodology (lead); visualization (equal); writing – original draft (lead); writing – review and editing (supporting). **C. Skema:** Conceptualization (equal); formal analysis (supporting); methodology (supporting); resources (lead); supervision (lead); visualization (equal); writing – review and editing (lead).

## CONFLICT OF INTEREST STATEMENT

The authors declare no conflict of interest.

## Supporting information


Video S1

Video S2

Appendix S1

## Data Availability

Data are available from Dryad: https://doi.org/10.5061/dryad.1vhhmgqxs.
