# Supplementary material for: Just add water: Rainfall‐induced anther closure and color change in Ripariosida hermaphrodita (Malvaceae)
Source: Ecol Evol. 2023 Jul 3;13(7):e10219. doi: 10.1002/ece3.10219 (PMC10316374; doi:10.1002/ece3.10219)
Supplement: Supplementary file 3 — Appendix S1 [file ECE3-13-e10219-s002.docx]

**Time-Lapse Legends**

**Video S1** Time-Lapse A. *Ripariosida hermaphrodita* anther closure and color change over 22:30 minutes. Flowers were misted with water every five seconds for the first eleven minutes.

**Video S2** Time-Lapse B. *Ripariosida hermaphrodita* anther closure and color change over 67:30 minutes. Flowers were misted with water every five seconds for the first ten minutes.
